# Associations of sedentary behavior and weekday lifestyle with mental health among Chinese senior high school students

**DOI:** 10.3389/fpsyg.2026.1855620

**Published:** 2026-07-03

**Authors:** Rongchang Fan, Kaibin Fan

**Affiliations:** 1 Department of Physical Education, Nanjing Railway College, Nanjing, Jiangsu, China; 2 School of Sports Training, Nanjing Sport Institute, Nanjing, Jiangsu, China

**Keywords:** adolescent mental health, dietary habits, lifestyle, sedentary behavior, senior high school students

## Abstract

**Background:**

Students in senior high school face substantial academic demands, prolonged sedentary time, constrained daily routines, and multiple imbalances in health-related behaviors. Their mental health may therefore be shaped by dietary habits, lifestyle, sedentary behavior, physical activity level, and body mass index (BMI). However, within the same school context, it remains unclear which factors—dietary habits, lifestyle habits, sedentary behavior, physical activity level, or BMI—show stronger associations with students' mental health.

**Methods:**

This cross-sectional questionnaire study was based on a school-based questionnaire survey conducted among students aged 15–17 years from a senior high school in Nanjing, China. Mental health was assessed using the World Health Organization-Five Wellbeing Index (WHO-5). Data on dietary habits, lifestyle, sedentary behavior, physical activity level, and BMI were collected. Correlation analyses and multiple linear regression analyses were performed to examine the associations between these behavioral factors and mental health.

**Results:**

Overall mental health in the sample was moderate. Sedentary time was prolonged, physical activity level was generally low, and dietary patterns were characterized by relatively regular main meals but suboptimal supplementary dietary habits. Correlation analyses showed that mental health was positively correlated with overall dietary habits (*r* = 0.243, *p* < 0.01) and overall lifestyle habits (*r* = 0.279, *p* < 0.01), and negatively correlated with sedentary habits (*r* = −0.756, *p* < 0.01), with sedentary habits showing the strongest correlation. In the overall multivariable model, physical activity level was negatively associated with mental health (β = −0.330, *p* < 0.001), a finding that should be interpreted cautiously because of the cross-sectional design and measurement approach; BMI category was also negatively associated (β = −0.148, *p* < 0.001), whereas overall dietary habits (β = 0.167, *p* < 0.001) and overall lifestyle habits (β = 0.248, *p* < 0.001) were positively associated with mental health. In the dimension-specific model, sedentary habits showed the strongest negative association (β = −0.722, *p* < 0.001), whereas weekday lifestyle habits (β = 0.168, *p* < 0.001) and regular meal habits (β = 0.074, *p* = 0.011) were positive correlates. Supplementary dietary habits (β = 0.050, *p* = 0.080) and weekend lifestyle habits (β = 0.025, *p* = 0.421) were not independently significant.

**Conclusion:**

Mental health in senior high school students was closely related to the structure of daily behaviors, with sedentary behavior showing the most prominent adverse association. Compared with focusing solely on increasing exercise, reducing sedentary behavior, optimizing weekday routines, and maintaining regular main meals may better align with the mental health promotion needs of the school setting.

## 1 Introduction

Adolescent mental health is not a peripheral issue but a central public health concern characterized by early onset, substantial burden, and long-term consequences. A systematic review and meta-analysis showed that approximately one in five children and adolescents worldwide experience depression or depressive symptoms, and that this burden is increasing (Lu et al., 2024). Another large-scale meta-analysis of epidemiological studies reported that the peak age at onset for any mental disorder is approximately 14.5 years, with 34.6% of cases occurring before age 14 and 48.4% before age 18 (Solmi et al., 2022), indicating that the later secondary school years represent a critical window for the identification of and intervention in mental health problems. Evidence from China likewise underscores the urgency of this issue. A clinical diagnostic epidemiological survey based on 17,524 children and adolescents reported a weighted prevalence of 17.5% for any mental disorder among those aged 6–16 years (Li F. et al., 2022). A 2024 systematic review further found that the pooled prevalence of depressive symptoms among Chinese children and adolescents was 26.17%, with the highest prevalence observed among senior high school students at 28.23% (Zhou et al., 2024). For students in senior high school, this risk is further compounded by the school context. Among the 52 studies included in a systematic review of academic pressure, the vast majority reported positive associations between academic pressure and outcomes such as depression, anxiety, self-harm, or suicidality (Steare et al., 2023). Mental health problems in senior high school students therefore need to be understood against the combined background of academic load and daily behavioral structure.

Existing research has suggested that, in 2016, 81.0% of school-going adolescents aged 11–17 years worldwide did not meet the recommended level of physical activity (Guthold et al., 2020). The World Health Organization (WHO) evidence review on physical activity and sedentary behavior in children and adolescents further indicated that higher levels of physical activity and restriction of sedentary behavior are associated with better health outcomes (Chaput et al., 2020). In relation to mental health, a longitudinal systematic review and meta-analysis found that higher sedentary behavior was associated with subsequent increases in depression, anxiety, and other mental health problems, with evidence of a dose–response relationship (Zhang et al., 2022). A systematic review in adolescents similarly indicated that excessive screen exposure is associated with poorer mental health (Santos et al., 2023). At the same time, sleep and mental health are bidirectionally related; shorter sleep duration, poorer sleep quality, greater insomnia symptoms, and chronotype may interact with internalizing problems, externalizing problems, and lower psychological wellbeing (Bacaro et al., 2024). In the present study, sleep duration was included within the lifestyle habits scale, but sleep quality and chronotype were not assessed as separate covariates. Regarding physical activity, the most recent systematic review and meta-analysis supports the role of physical activity interventions in improving anxiety, depression, stress, self-esteem, and social competence, although the evidence remains highly heterogeneous and its independent role after joint adjustment for other behaviors remains insufficiently clear (Fu et al., 2025). In terms of diet, breakfast skipping is associated with increased risks of depression, psychological distress, and anxiety in adolescents (Zahedi et al., 2022), whereas healthy dietary patterns are associated with lower risk of depression (Zahedi et al., 2022). A meta-analysis of cohort studies also suggested that higher body mass index (BMI) is associated with an increased subsequent risk of depression (Chaplin et al., 2023). Taken together, these findings suggest that adolescent mental health is more likely to reflect the cumulative exposure of multiple lifestyle behaviors rather than the isolated effect of any single factor.

Several gaps in the current evidence remain highly relevant to school practice. First, diet, physical activity, sedentary behavior, sleep, and screen-related behaviors are often studied in isolation. Although systematic reviews have indicated that these behaviors tend to cluster into healthy, unhealthy, or mixed patterns and are associated with poorer physical and mental outcomes (Alosaimi et al., 2023), studies that simultaneously incorporate multiple behavioral domains within a single analytical framework and compare their relatively independent associations remain limited. Second, the operationalization of dietary variables has focused largely on overall diet quality or ultra-processed foods, whereas evidence on regular meal patterns, a measure more directly aligned with everyday school management, remains relatively limited (Zahedi et al., 2022). Third, sedentary behavior has often been treated simply as screen time, with little differentiation among classroom sitting, after-school homework, recreational screen use, and the compression of daily routines that together shape school-based lifestyles (Alosaimi et al., 2023). Fourth, although Chinese studies have suggested a dose–response relationship between combined unhealthy lifestyle behaviors and the risk of depression (Zhang et al., 2024), most samples have covered children and adolescents as a whole. Comprehensive analyses simultaneously examining dietary habits, lifestyle, sedentary behavior, physical activity, and BMI categories among general senior high school students within an East Asian school context remain insufficient.

Against this background, the present study focused on students attending a general senior high school in Nanjing and examined the associations of mental health with dietary habits, lifestyle, sedentary behavior, physical activity level, and BMI categories. Rather than considering a single behavior in isolation, this study sought to place regular meal patterns, weekday and weekend lifestyles, sedentary habits, and physical activity level within the same analytical framework to examine their associations with mental health and their relatively independent roles. We hypothesized that healthier dietary and lifestyle habits and higher physical activity levels would be positively associated with mental health, whereas sedentary habits and higher BMI categories would be negatively associated with mental health.

## 2 Methods

### 2.1 Study design and participants

This study used a cross-sectional questionnaire design. The survey was conducted during a regular school semester among senior high school students aged 15–17 years enrolled at Nanjing No. 12 High School. A school-based convenience sampling method was used. With the assistance of school administrators and class teachers, students from existing classes were invited to participate voluntarily. Students were eligible if they were enrolled in the participating school, were aged 15–17 years, were able to understand and complete the questionnaire independently, and provided informed assent together with parental or guardian consent. Students were excluded if they did not provide consent, submitted incomplete questionnaires, had missing data on key variables, or provided responses with clear logical inconsistencies. The questionnaire covered modules on basic personal information, dietary habits, lifestyle habits, physical activity, and mental health. A total of 600 questionnaires were distributed, and 550 were returned, yielding a response rate of 91.7%. After collection, all questionnaires were checked for completeness, missing values in key variables, and logical consistency according to the predefined inclusion and exclusion criteria. Questionnaires with incomplete key information, missing responses in major study variables, or clear logical inconsistencies were excluded before analysis. Questionnaires were excluded only when they had incomplete key information, missing responses in major study variables, or logically inconsistent physical activity data. Reporting 0 days of physical activity was not treated as an exclusion criterion; students with complete and logically consistent responses but no reported physical activity were retained and classified as having low physical activity. A total of 512 valid questionnaires were ultimately included, with an effective rate of 93.1%. After data cleaning, the final analytic sample included only questionnaires with complete data for all variables used in the analyses, including sex, age, height, weight, waist circumference, BMI, dietary habits, lifestyle habits, physical activity variables, and WHO-5 items. Therefore, no missing values remained in the final dataset for the variables included in the correlation and regression analyses.

Because this was a school-based convenience sample, all eligible and consenting students in the participating classes were invited to participate. The adequacy of the final sample size was evaluated using G^*^Power 3.1.9.4 based on the main multiple linear regression analysis. The parameters were set as follows: *F* tests, linear multiple regression fixed model, *R*^2^ deviation from zero, medium effect size *f*^2^ = 0.15, α = 0.05, statistical power = 0.95, and eight predictors. The minimum required sample size was 160. The final analytic sample of 512 students exceeded this requirement.

### 2.2 Instruments and variable measurement

#### 2.2.1 Basic demographic and anthropometric indicators

The basic information section included sex, age, height, weight, and waist circumference. Body mass index (BMI) was calculated from height and weight using the formula weight (kg)/height (m)^2^. BMI was classified into four categories: underweight, normal weight, overweight, and obesity, according to the Chinese age- and sex-specific BMI screening standards for school-age children and adolescents (Simpson et al., 2020). BMI ≥28.0 was defined as obesity, and 24.0–27.9 as overweight. For boys, 17.9–23.9 was classified as normal weight; for girls, 17.2–23.9 was classified as normal weight. Values below the corresponding normal range were classified as underweight. Participants who did not meet the criteria for high or moderate physical activity, including those reporting no physical activity during the previous week, were classified as having low physical activity if their physical activity responses were otherwise complete and logically consistent.

#### 2.2.2 Assessment of dietary habits

Dietary habits were assessed using a study-specific Dietary Habits Scale constructed from commonly used adolescent dietary behavior items. These items covered regular meal consumption and food-frequency indicators similar to those used in international school-based adolescent health surveys, including breakfast, fruit, vegetables, sugar-sweetened beverages, sweets, and fast food (Inchley et al., 2018; Timor, 2018). The recall period was the previous week. The scale comprised two dimensions: regular meal habits and supplementary dietary habits. Regular meal habits reflected the regularity of breakfast, lunch, and dinner. Supplementary dietary habits included the frequency of fruit, vegetables, milk, sugar-sweetened beverages, fast food, instant noodles or ramen, and sweets. Each item was assigned an ordinal score according to frequency or number of days. Unhealthy dietary items, including sugar-sweetened beverages, fast food, instant noodles or ramen, and sweets, were reverse-coded so that higher scores consistently indicated more regular or healthier dietary habits. No predefined clinical or diagnostic cut-off values were applied. The dimension scores and overall dietary habits score were calculated as mean scores and analyzed as continuous variables. Exploratory factor analysis and Cronbach's α were used to examine the structural validity and internal consistency of the scale in the present sample.

#### 2.2.3 Assessment of lifestyle and sedentary behavior

Lifestyle and sedentary behavior were assessed using a study-specific Lifestyle Habits Scale constructed from commonly used adolescent health behavior indicators, including leisure screen time, sedentary leisure activities, homework/study/reading time, parental restriction of screen use, and sleep duration. These domains are consistent with behavioral indicators commonly used in adolescent sedentary behavior and daily lifestyle research (Burtscher et al., 2023; Craig et al., 2003). The scale was not used as a clinical diagnostic instrument. It comprised three dimensions: weekday lifestyle habits, weekend lifestyle habits, and sedentary habits. Weekday and weekend lifestyle habits included leisure screen exposure time, time spent sitting while listening to music, time spent sitting while talking on the phone, time spent on homework/studying/reading, parental restriction on screen time, and sleep duration. Sedentary habits reflected preference for sedentary activities and tendencies related to reducing sedentary behavior. Items were coded according to the study protocol before mean scores were calculated. Higher scores for weekday and weekend lifestyle habits indicated healthier daily routines, whereas higher scores for sedentary habits indicated a stronger tendency toward sedentary behavior. No predefined clinical or diagnostic cut-off values were applied. The dimension scores and overall lifestyle score were calculated as mean scores and analyzed as continuous variables. Exploratory factor analysis and Cronbach's α were used to examine the structural validity and internal consistency of the scale in the present sample.

#### 2.2.4 Assessment of physical activity

Physical activity was assessed using items adapted from the short form of the International Physical Activity Questionnaire (IPAQ). The items assessed the frequency and average daily duration of vigorous physical activity, moderate physical activity, walking, and sitting time during the previous week. Weekly metabolic equivalent of task minutes (MET-min/week) were calculated using the metabolic equivalent coefficients for activities of different intensities according to the formula: MET coefficient × minutes of activity/day × days/week. The MET coefficients for walking, moderate-intensity activity, and vigorous-intensity activity were 3.3, 4.0, and 8.0, respectively. Based on weekly MET-min and the number of activity days, physical activity level was classified into three categories: high, moderate, and low. High physical activity level was defined as meeting either of the following criteria: vigorous activity on at least 3 days accumulating no less than 1500 MET-min/week, or any combination of walking, moderate-intensity activity, and/or vigorous-intensity activity on 7 days accumulating at least 3000 MET-min/week. Moderate physical activity level was defined as meeting any of the following criteria: vigorous activity on ≥3 days for at least 20 min/day, or moderate-intensity activity and/or walking on ≥5 days for at least 30 min/day, or any combination of walking, moderate-intensity activity, and/or vigorous-intensity activity on ≥5 days accumulating at least 600 MET-min/week. Those who did not meet these criteria were classified as having low physical activity. Physical activity level was entered into the analyses as an ordinal categorical variable, and sitting time was described in min/day (Craig et al., 2003; Li et al., 2010). The full IPAQ was not administered as a separate questionnaire because the present school-based survey also included dietary habits, lifestyle habits, sedentary behavior, anthropometric indicators, and mental health, and the questionnaire length needed to be appropriate for classroom-based administration. Therefore, an adapted physical activity module was used, while the IPAQ-based MET coefficients and physical activity classification criteria were retained (Craig et al., 2003; Fogelholm et al., 2006).

#### 2.2.5 Assessment of mental health

Mental health was assessed using the World Health Organization Five Wellbeing Index (WHO-5), a widely used brief measure of subjective psychological wellbeing, with a recall period of the previous 2 weeks (Topp et al., 2015; de Wit et al., 2007). The scale contains five items and uses a five-point Likert response format. The mean score of the scale was calculated as the indicator of mental health, with higher scores indicating better mental health. Exploratory factor analysis and Cronbach's α coefficient testing were performed for this scale in the present sample.

#### 2.2.6 Scale reliability and validity testing

To ensure the stability of measurement in the present sample, structural validity and internal consistency were examined separately for the Dietary Habits Scale, the Lifestyle Habits Scale, and the WHO-5 (Topp et al., 2015). Structural validity was assessed using exploratory factor analysis. The Kaiser–Meyer–Olkin (KMO) test and Bartlett's test of sphericity were used to evaluate sampling adequacy. Principal component analysis was used to extract common factors, followed by varimax rotation. Internal consistency was evaluated using Cronbach's α coefficient (de Wit et al., 2007).

### 2.3 Statistical Analysis

All questionnaire data were coded according to uniform scoring rules before analysis. All statistical analyses were performed using SPSS 26.0. BMI category and physical activity level were treated as ordinal categorical variables in the correlation and regression analyses, whereas waist circumference and scale scores were treated as continuous variables. Spearman rank correlation analysis was used for ordinal variables, and Pearson correlation analysis was used for continuous variables. Categorical variables were described using frequencies and constituent ratios, and continuous variables were described using means ± standard deviations; minimum and maximum values were reported when necessary. Structural validity of the scales was assessed using exploratory factor analysis, and internal consistency was evaluated using Cronbach's α coefficient. In the group comparisons, differences in sex, age, and BMI category across physical activity levels (Li et al., 2010) were tested using the chi-square test. Differences in waist circumference, dietary habits, lifestyle habits, and mental health across physical activity levels were examined using one-way analysis of variance. Differences in mental health by sex were examined using the independent-samples *t* test, whereas differences in mental health by age and BMI category were examined using one-way analysis of variance. When the overall difference was statistically significant, *post hoc* multiple comparisons were conducted. Different methods were used for correlation analyses according to variable type. Spearman rank correlation analysis was used for ordinal variables, including physical activity level, BMI category, and age. Pearson correlation analysis was used for continuous variables, including scores for dietary habits, lifestyle habits, sedentary habits, the mental health scale, and waist circumference.

Multivariable analyses were performed using multiple linear regression models, with the mean score of the mental health scale as the dependent variable. In the overall model, sex, age, height, and waist circumference were first entered as control variables, followed by BMI category, physical activity level, overall dietary habits, and overall lifestyle, to examine the independent associations of these variables with mental health. During model fitting, multicollinearity diagnostics were performed, and variables with a variance inflation factor (VIF) greater than 10 were excluded. The overall model was then refitted after removing height and waist circumference. To further compare the independent roles of specific behavioral dimensions, an additional set of multiple linear regression models was established in which regular meal habits, supplementary dietary habits, weekday lifestyle habits, weekend lifestyle habits, and sedentary habits were entered simultaneously. Regression results are reported as unstandardized regression coefficients (B), standard errors, standardized regression coefficients (β), *t* values, *P* values, model *R*, adjusted *R*^2^, *F* values, and VIF. All tests were two-sided, and *P* < 0.05 was considered statistically significant.

### 2.4 Ethics statement

This study was implemented after review and approval by the ethics committee. All participants took part in the survey on the basis of informed consent. Given that the sample comprised minors, informed consent was obtained from both the students and their guardians before the survey. The questionnaire data were used only for research analyses, and personal information was kept confidential throughout the study.

## 3 Results

### 3.1 Characteristics of the study participants

A total of 512 senior high school students were included in this study, with a broadly balanced sex distribution: 252 boys (49.2%) and 260 girls (50.8%). Participants were aged 15–17 years, with all age groups represented. The largest proportion was 16 years old (39.3%), followed by 15 years (35.0%) and 17 years (25.8%). Anthropometric indicators were generally within expected ranges, with a mean height of 166.66 cm (154.1–183.5), a mean weight of 56.68 kg (40.8–84.6), and a waist circumference of 70.81±4.77 cm (63.05–80.75). BMI categories showed that most students were within the normal weight range (66.8%), although a proportion had abnormal weight status, including underweight (21.9%), overweight (8.4%), and obesity (2.9%). As shown in Table 1, the sample was relatively balanced in terms of sex and age structure, and overall physical development was within the normal range, although some variation in weight status was observed, providing a basis for subsequent analyses of weight-related factors and mental health.

**Table 1:** Participant characteristics of senior high school students.

Variable	Category/statistic	*n*	%	Value
**Sex**	Male	252	49.2	–
	Female	260	50.8	–
**Age**	15 years	179	35	–
	16 years	201	39.3	–
	17 years	132	25.8	–
**Height (cm)**	Overall	–	–	Mean = 166.66; range = 154.1– 183.5
**Weight (kg)**	Overall	–	–	Mean = 56.68; range = 40.8– 84.6
**BMI category**	Underweight	112	21.9	–
	Normal weight	342	66.8	–
	Overweight	43	8.4	–
	Obesity	15	2.9	–
**Waist circumference (cm)**	Overall	512	100	70.81 ± 4.77; range = 63.05– 80.75

BMI, body mass index. Values are presented as *n* (%), mean, range, or mean ± standard deviation as appropriate.

### 3.2 Structural validity and internal consistency of the measures

All scales demonstrated good indicators of structural validity (Table 2). The KMO values for the Dietary Habits Scale, the Lifestyle Habits Scale, and the World Health Organization Five Wellbeing Index (WHO-5) were 0.878, 0.898, and 0.908, respectively, and Bartlett's test of sphericity was significant for all three scales (all *p* < 0.001), indicating that the data were suitable for structural validity testing. The cumulative variance explained by the three scales was 69.573%, 69.001%, and 79.377%, respectively, suggesting a stable overall dimensional structure. Internal consistency analysis showed that Cronbach's α for the total scores of the three scales was 0.843, 0.859, and 0.935, respectively, all at acceptable levels. Cronbach's α for the subscales ranged from 0.832 to 0.914. For the Dietary Habits Scale, the values were 0.904 for regular meal habits and 0.900 for supplementary dietary habits. For the Lifestyle Habits Scale, the values were 0.877 for weekday lifestyle habits, 0.914 for weekend lifestyle habits, and 0.832 for sedentary habits. Overall, the scales used in this study showed good structural validity and internal consistency.

**Table 2:** Structural validity and internal consistency of the study measures.

Measure	Subscales	KMO	Bartlett's *χ^2^*	df	*p* value	Cumulative variance explained (%)	Cronbach's α (total)	Cronbach's α (subscales)
Dietary habits scale	Regular meal habits; supplementary dietary habits	0.878	2,949.624	45	< 0.001	69.573	0.843	Regular meal habits = 0.904; supplementary dietary habits = 0.900
Lifestyle habits scale	Weekday lifestyle habits; weekend lifestyle habits; sedentary habits	0.898	3,718.991	91	< 0.001	69.001	0.859	Weekday lifestyle habits = 0.877; weekend lifestyle habits = 0.914; sedentary habits = 0.832
WHO-5 scale	Single-factor structure	0.908	2,067.717	10	< 0.001	79.377	0.935	–

### 3.3 Descriptive characteristics of dietary habits, lifestyle behaviors, sedentary time, physical activity, and mental health

In terms of dietary behaviors, some variation was observed in the regularity of the three main meals (Table 3). Breakfast and dinner were most commonly consumed on 3–5 days/week, whereas lunch was most commonly consumed on 6–7 days/week, suggesting that lunch intake was relatively more stable. For supplementary dietary behaviors, fruit intake and vegetable intake with meals were most commonly reported on 4–5 days/week, and milk intake was most commonly reported on 6–7 days/week; however, 77 students (15.0%) reported not drinking milk. Sugar-sweetened beverages, instant noodles, and sweets were most commonly consumed on 2–3 days/week, whereas fast food was most commonly consumed on 4–5 days/week. Lifestyle behaviors showed that on weekdays, leisure screen time was most commonly 1–2 h/d, sitting while listening to music and time spent on homework/studying/reading were both mainly 2–3 h/d, sitting while talking on the phone was most commonly 1–1.5 h/d, and sleep duration was mainly concentrated at 5–6 h/d. On weekends, leisure screen time and sitting while listening to music were both most commonly reported as more than 4 h/d, sitting while talking on the phone remained most commonly 1–1.5 h/d, homework/studying/reading was mainly 2–3 h/d, and sleep duration was most commonly 6–7 h/d. With regard to sedentary-related behaviors, both preference for sedentary activities and the tendency to encourage family members to reduce sedentary behavior were mainly at a moderate level.

**Table 3:** Descriptive statistics of key behavioral variables.

Domain	Indicator	Main finding
Regular meal habits	Breakfast	The largest proportion reported eating breakfast on 3–5 days/week, *n* = 175 (34.2%)
	Lunch	The largest proportion reported eating lunch on 6–7 days/week, *n* = 167 (32.6%)
	Dinner	The largest proportion reported eating dinner on 3–5 days/week, *n* = 179 (35.0%)
Supplementary dietary habits	Fruit intake	Most students reported fruit intake on 4–5 days/week, *n* = 206 (40.2%)
	Sugar-sweetened beverages	Most students reported intake on 2–3 days/week, *n* = 179 (35.0%)
	Fast food	Most students reported intake on 4–5 days/week, *n* = 167 (32.6%)
	Instant noodles/ramen	Most students reported intake on 2–3 days/week, *n* = 152 (29.7%)
	Sweets	Most students reported intake on 2–3 days/week, *n* = 146 (28.5%)
	Vegetables with meals	Most students reported intake on 4–5 days/week, *n* = 159 (31.1%)
	Milk	The largest proportion reported intake on 6–7 days/week, *n* = 145 (28.3%); 77 students (15.0%) reported no milk intake
Weekday lifestyle habits	Leisure screen time	1–2 h/day was most common, *n* = 159 (31.1%)
	Sitting while listening to music	2–3 h/day was most common, *n* = 182 (35.5%)
	Sitting while talking on the phone	1–1.5 h/day was most common, *n* = 168 (32.8%)
	Homework/study/reading time	2–3 h/day was most common, *n* = 165 (32.2%)
	Sleep duration	5–6 h/day was most common, *n* = 172 (33.6%)
Weekend lifestyle habits	Leisure screen time	More than 4 h/day was most common, *n* = 152 (29.7%)
	Sitting while listening to music	More than 4 h/day was most common, *n* = 141 (27.5%)
	Sitting while talking on the phone	1–1.5 h/day was most common, *n* = 145 (28.3%)
	Homework/study/reading time	2–3 h/day was most common, *n* = 138 (27.0%)
	Sleep duration	6–7 h/day was most common, *n* = 142 (27.7%)
Sedentary habits	Preference for sedentary activities	“Moderately like” was most common, *n* = 191 (37.3%)
	Encouraging family members to reduce sedentary behavior	“Moderately encourage” was most common, *n* = 170 (33.2%)
Sitting time	Daily sitting time	442.91 ± 95.97 min/day
Physical activity level	Distribution	Low = 256 (50.0%); Moderate = 178 (34.8%); High = 78 (15.2%)
Psychological health	Overall WHO-5 profile	Overall psychological health was generally moderate

For a journal submission, this is the most concise version. If you want, I can also expand this into a full supplementary descriptive table.

Overall, mean sitting time was 442.91±95.97 min/d. The distribution of physical activity levels showed that 50.0% of students were in the low category, 34.8% in the moderate category, and 15.2% in the high category. WHO-5 results indicated that overall mental health in the sample was at a moderate level.

### 3.4 Bivariate associations between mental health and key study variables

**Figure 1:**
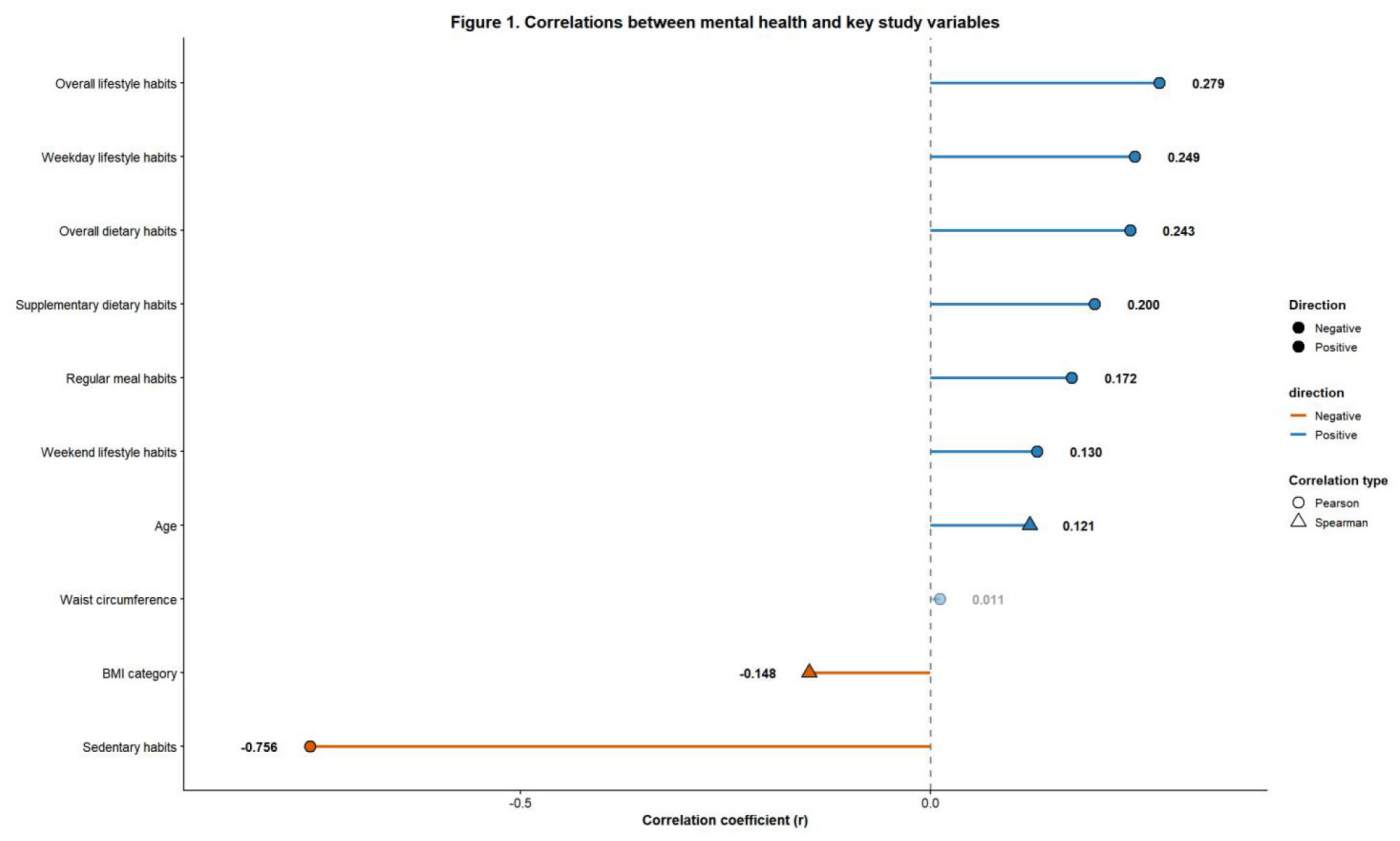
Correlations between mental health and key study variables. Bars indicate correlation coefficients between mental health and key study variables. Positive values indicate positive associations and negative values indicate negative associations.

Bivariate correlation analyses showed that mental health was positively correlated with age and negatively correlated with BMI category, whereas no significant correlation was observed with waist circumference (Table 4). In terms of dietary behaviors, overall dietary habits and both of its dimensions were positively correlated with mental health, with both regular meal habits and supplementary dietary habits reaching statistical significance. For lifestyle behaviors, overall lifestyle, weekday lifestyle, and weekend lifestyle were all positively correlated with mental health. Among all behavioral variables, sedentary habits showed the strongest correlation with mental health, with a significant negative correlation (*r* = −0.756, *p* < 0.01), and the strength of this correlation was markedly greater than that of the other variables. Overall, most dietary and lifestyle indicators were positively associated with mental health, whereas sedentary habits showed the strongest negative association, as illustrated in Figure 1.

**Table 4:** Correlations between psychological health and key study variables.

Variable	Method	*r*	*p* value	Direction
Age	Spearman	0.121	< 0.01	Positive
BMI category	Spearman	−0.148	< 0.01	Negative
Waist circumference	Pearson	0.011	>0.05	Not significant
Overall dietary habits	Pearson	0.243	< 0.01	Positive
Regular meal habits	Pearson	0.172	< 0.01	Positive
Supplementary dietary habits	Pearson	0.2	< 0.01	Positive
Overall lifestyle habits	Pearson	0.279	< 0.01	Positive
Weekday lifestyle habits	Pearson	0.249	< 0.01	Positive
Weekend lifestyle habits	Pearson	0.13	< 0.01	Positive
Sedentary habits	Pearson	−0.756	< 0.01	Strong negative

BMI, body mass index. Higher sedentary habit scores were strongly associated with lower psychological health scores in this dataset.

### 3.5 Independent associations of overall behavioral factors with mental health

Multiple linear regression analysis showed that, after sex, age, BMI category, physical activity level, overall dietary habits, and overall lifestyle were entered simultaneously into the model, age, BMI category, physical activity level, overall dietary habits, and overall lifestyle were all independently associated with mental health, whereas sex was not statistically significant (Table 5A). Specifically, age was positively associated with mental health (β = 0.084, *p* = 0.030), whereas BMI category (β = −0.148, *p* < 0.001) and physical activity level (β = −0.330, *p* < 0.001) were negatively associated with mental health. Overall dietary habits (β = 0.167, *p* < 0.001) and overall lifestyle habits (β = 0.248, *p* < 0.001) were positively associated with mental health. Based on the standardized regression coefficients, physical activity level showed the strongest association, followed by overall lifestyle, overall dietary habits, and BMI category. The model as a whole was statistically significant (*F* = 30.168, *p* < 0.001), with an adjusted *R*^2^ of 0.255, indicating that it explained 25.5% of the variance in mental health. VIF values ranged from 1.019 to 1.128.

**Table 5A:** Multiple linear regression of psychological health on overall behavioral variables.

Variable	*B*	SE	β	*t*	*p* value	VIF
Sex	−0.174	0.117	−0.06	−1.48	0.14	1.128
Age	0.157	0.072	0.084	2.176	0.03	1.022
BMI category	−0.333	0.088	−0.148	−3.788	< 0.001	1.048
Physical activity level	−0.656	0.079	−0.33	−8.269	< 0.001	1.091
Overall dietary habits	0.292	0.069	0.167	4.254	< 0.001	1.055
Overall lifestyle habits	0.479	0.074	0.248	6.442	< 0.001	1.019

Model fit: *R* = 0.514; adjusted *R*^2^ = 0.255; *F* = 30.168, *p* < 0.001. *B*, unstandardized coefficient; SE, standard error; β, standardized coefficient; VIF, variance inflation factor.

### 3.6 Independent associations of specific dietary and lifestyle dimensions with mental health

**Figure 2:**
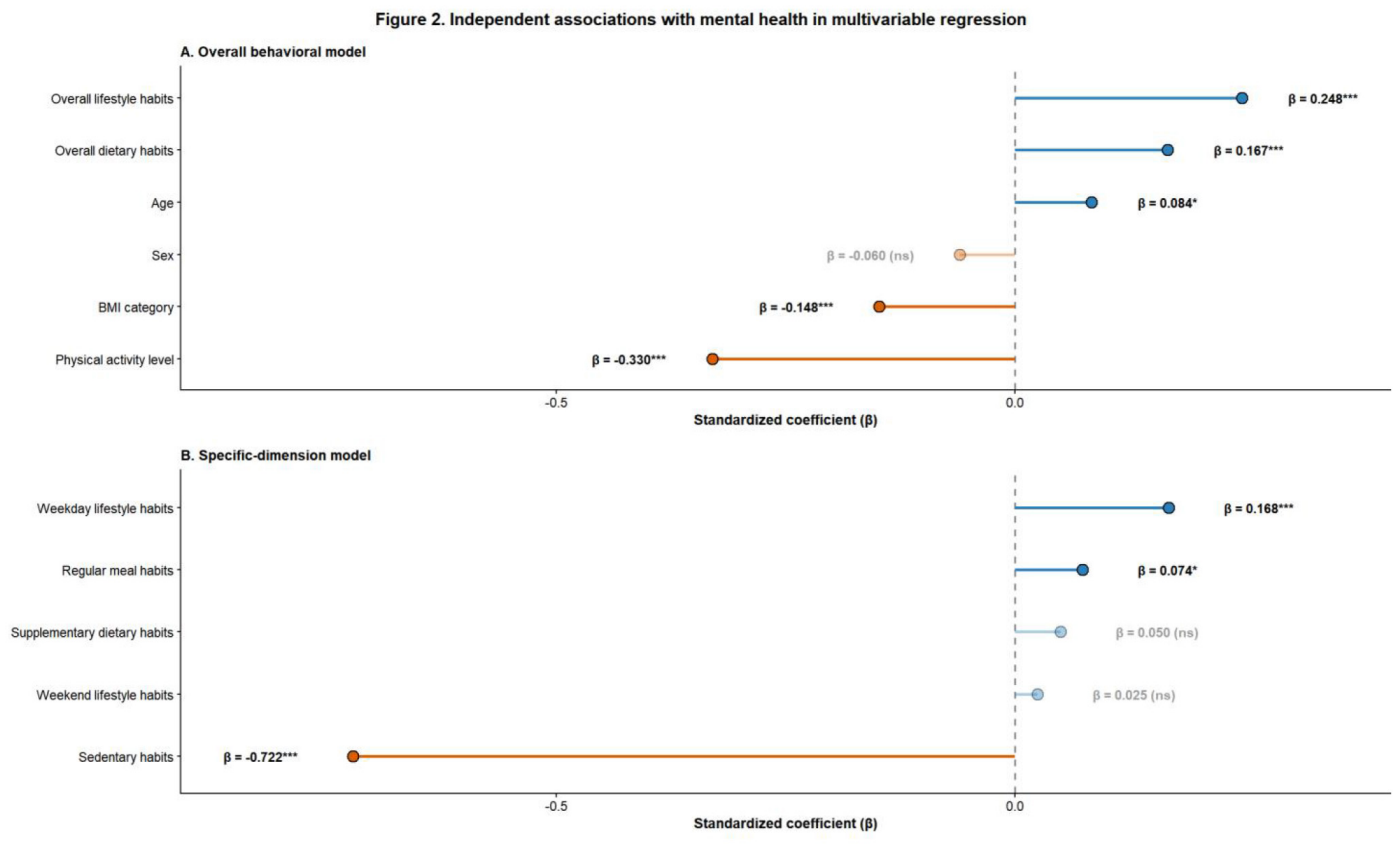
Independent associations with mental health in multivariable regression Panels show standardized regression coefficients from the overall behavioral model and the specific-dimension model. Positive β values indicate positive associations with mental health, whereas negative β values indicate negative associations. **(A)** Overall behavioral model. **(B)** Specific-dimension model.

When the dimensions of diet and lifestyle were entered simultaneously into the multiple linear regression model, regular meal habits, weekday lifestyle habits, and sedentary habits were all independently associated with mental health, whereas supplementary dietary habits and weekend lifestyle habits were not statistically significant (Table 5B). Specifically, regular meal habits were positively associated with mental health (β = 0.074, *p* = 0.011), weekday lifestyle habits were positively associated with mental health (β = 0.168, *p* < 0.001), and sedentary habits were negatively associated with mental health (β = −0.722, *p* < 0.001). In terms of standardized regression coefficients, sedentary habits had the largest absolute value, indicating that it was the strongest independent correlate of mental health among all dimensions, as shown in Figure 2. By contrast, supplementary dietary habits (β = 0.050, *p* = 0.080) and weekend lifestyle habits (β = 0.025, *p* = 0.421) were not significantly associated with mental health in the model. The model as a whole was statistically significant (*F* = 160.382, *p* < 0.001), with an adjusted *R*^2^ of 0.609, indicating that it explained 60.9% of the variance in mental health. VIF values ranged from 1.041 to 1.278.

**Table 5B:** Multiple linear regression of psychological health on specific dietary and lifestyle dimensions.

Variable	*B*	SE	β	*t*	*p* value	VIF
Regular meal habits	0.112	0.044	0.074	2.556	0.011	1.1
Supplementary dietary habits	0.066	0.038	0.05	1.757	0.08	1.041
Weekday lifestyle habits	0.269	0.049	0.168	5.52	< 0.001	1.219
Weekend lifestyle habits	0.034	0.042	0.025	0.806	0.421	1.278
Sedentary habits	−1.035	0.041	−0.722	−25.394	< 0.001	1.056

Model fit: *R* = 0.783; adjusted *R*^2^ = 0.609; *F* = 160.382, *p* < 0.001. *B*, unstandardized coefficient; SE, standard error; β, standardized coefficient; VIF, variance inflation factor.

## 4 Discussion

This study showed that mental health among senior high school students was generally at a moderate level, with prolonged sedentary time, a high proportion of low physical activity, and dietary behaviors characterized by relatively regular main meals but room for improvement in supplementary dietary habits. Correlation analyses suggested that mental health was positively associated with overall dietary habits and lifestyle habits and negatively associated with sedentary habits. In the multivariable models, sedentary habits emerged as the strongest independent correlate, whereas weekday lifestyle habits and regular meal habits retained stable positive associations. These findings suggest that mental health in senior high school is not simply a matter of insufficient exercise, but is also closely related to the overall structure of daily behaviors.

The present findings are broadly consistent with the existing evidence. A longitudinal systematic review and meta-analysis in children and adolescents showed that higher sedentary behavior is associated with increased subsequent depression, anxiety, and other mental health problems, with evidence of a dose–response relationship (Zhang et al., 2022). Systematic reviews, cohort meta-analyses, and prospective studies focusing on screen time have likewise indicated that the association between screen exposure and poorer mental health is relatively consistent in adolescents, although the effect sizes are usually modest; the direction of association is stable, particularly for depressive symptoms (Makdissi et al., 2023; Li L. et al., 2022; Nagata et al., 2024). More importantly, sedentary behavior is not equivalent to insufficient physical activity. Cohort studies in adolescents based on objective measurement have shown that greater sedentary time and lower light physical activity are associated with higher depressive symptoms, and screen-based sedentary behavior often shows a more stable adverse association than total sitting time (Opdal et al., 2020; Kandola et al., 2020; Wilhite et al., 2023). The longitudinal study by Boers et al. further suggested that the link between social media use and depressive symptoms is more consistent with upward social comparison and a reinforcing spiral mechanism than with the simple replacement of time spent on exercise (Boers et al., 2019). In the senior high school context, this finding is not unexpected. Classroom sitting, after-school homework, tutoring, and recreational screen exposure accumulate, making sedentary behavior a recurrent, sustained, and difficult-to-recover-from daily exposure. Its association with mental health may therefore be stronger than that of any single behavioral indicator.

Weekday lifestyle habits remained significant in the multivariable model, whereas weekend lifestyle habits did not, suggesting that for senior high school students, the exposure more closely resembling a chronic pattern may lie in the highly repetitive rhythms of weekdays rather than in short-term weekend fluctuations. A systematic review in adolescents showed that academic pressure is generally positively associated with mental health problems such as depression and anxiety (Steare et al., 2023). At the same time, research on 24-h behaviors has repeatedly shown that the combination of sufficient sleep, less screen time/sedentary behavior, and appropriate physical activity corresponds more consistently to better mental health outcomes than meeting any single behavioral recommendation alone. In this framework, screen time and sleep sometimes contribute to mental health to a degree not weaker than physical activity itself (Wilhite et al., 2023; Sampasa-Kanyinga et al., 2020; Loewen et al., 2019). Given the insufficient weekday sleep and extended study-related sitting time observed in the present sample, weekday routines, recovery time, and screen management may constitute a more stable behavioral foundation for mental health in senior high school students, whereas the independent role of weekend behaviors may be more easily attenuated in multivariable models because of their greater heterogeneity.

The finding that regular meal habits, rather than supplementary dietary habits, remained significant also has a fairly clear implication. A meta-analysis of breakfast studies showed that skipping breakfast is associated with increased risks of depression, psychological distress, and anxiety during adolescence (Zahedi et al., 2022). A prospective study found that higher frequencies of family breakfast and dinner were associated with lower subsequent incidence of common mental disorders (Agathão et al., 2021). In a Canadian adolescent sample, breakfast skipping was also associated with more psychosomatic symptoms (Peprah et al., 2024). These findings suggest that meal regularity is itself an important behavior related to mental health. For senior high school students, the rhythm of main meals is more directly connected to daytime energy supply, classroom functioning, and the stability of daily routines, which may explain why it retained an independent association in the combined model. By contrast, supplementary dietary behaviors such as fruit, milk, sugar-sweetened beverages, fast food, and snacks may reflect dietary structure, but these exposures are more fragmented, more context-dependent, and may share explanatory variance with other lifestyle variables; they therefore did not show stable significance in the multivariable model.

The negative association between BMI and mental health indicates that body composition remains a factor that should not be overlooked at this stage of development. Meta-analytic evidence has shown that overweight and obesity are associated with greater body dissatisfaction and lower self-esteem (Moradi et al., 2022). A UK birth cohort study further suggested that higher BMI may be linked to subsequent depressive symptoms through greater body dissatisfaction, with this pathway appearing more pronounced in girls (Blundell et al., 2024). This implies that abnormal BMI in senior high school is not merely a physical issue, but may also involve self-evaluation, appearance-based comparison, and weight-management pressure. From the perspective of school health promotion, mental health should not be considered separately from physical status.

The findings on physical activity require separate and cautious interpretation. In the present study, physical activity level was negatively associated with mental health, which was inconsistent with the generally expected protective association reported in most previous studies. Previous systematic reviews and meta-analyses have generally shown that higher physical activity or exercise participation is associated with better adolescent mental health, including lower levels of depression, anxiety, and psychological distress (Rodriguez-Ayllon et al., 2019; Ruiz-Ranz and Asín-Izquierdo, 2025; Marinelli et al., 2024). However, this association may vary across measurement approaches, activity contexts, and study designs. In a large adolescent study, Jussila et al. found a dose–response relationship between leisure-time moderate-to-vigorous activity and better mental health, whereas longer durations of active school travel were associated with higher depressive symptoms and help-seeking behavior (Jussila et al., 2023). This suggests that where activity occurs may be as important as how much activity is performed. On this basis, the unexpected direction of the physical activity finding in the present study may be related to the coding of activity levels, the inability of the scale to distinguish leisure activity from commuting or task-related activity, reverse causation in a cross-sectional design, and residual confounding such as academic burden that was not included in the model. A more cautious interpretation at this stage is therefore that physical activity was indeed associated with mental health in this study, but the direction and independent role of this association still require further verification in subsequent research, rather than being simplistically described as indicating that more physical activity is associated with poorer mental health.

The subgroup differences further suggested that mental health among senior high school students was not evenly distributed. In this study, mental health differed across sex, age, and BMI groups, with girls, older students, and those with normal BMI showing relatively better mental health. For school-based intervention, this implies that mental health promotion should not rely solely on uniform approaches, but should retain a degree of stratified perspective. Boys, younger students, and those with abnormal BMI may warrant greater attention in this sample. The age-related differences may reflect variation in grade adaptation, behavioral self-management, or psychological maturity, although this still requires confirmation through more fine-grained grade-level data and longitudinal research.

The practical significance of this study lies in shifting the focus of school mental health promotion from psychological counseling alone to the optimization of daily behavioral structure. For senior high school students, simply emphasizing “more exercise” is not sufficient. Reducing prolonged uninterrupted sitting, limiting nonessential screen exposure, protecting weekday sleep and recovery time, and maintaining regular breakfast, lunch, and dinner should likewise be incorporated into school health education and class management. This is consistent with the direction of existing evidence on academic pressure and combinations of multiple behaviors, suggesting that adolescent mental health is more likely to be shaped by a set of interwoven daily behaviors than by any single risk behavior alone (Zhang et al., 2024; Makdissi et al., 2023; Li L. et al., 2022; Nagata et al., 2024). In practice, schools may incorporate interruptions to sedentary time, activity during breaks, education on sleep and screen use, and prompts for regular meals into routine health promotion. At the family level, efforts should also support stable breakfast and dinner routines and provide more targeted support for students with abnormal BMI.

A strength of this study is its focus on the specific school context of senior high school, while simultaneously incorporating multiple variables, including dietary habits, lifestyle, sedentary habits, physical activity, and BMI, and using measurement tools with good structural validity and internal consistency, thereby allowing the associations between different behavioral dimensions and mental health to be compared within a single analytical framework. At the same time, this study has several limitations. First, the cross-sectional design precludes determination of temporal sequence and causal direction. Second, the behavioral variables were derived mainly from self-report questionnaires, making recall bias and social desirability bias difficult to avoid; the use of school-based convenience sampling from a single school also limits the generalizability of the findings. Third, although sleep duration was included within the lifestyle habits scale, sleep quality, insomnia symptoms, and chronotype were not assessed as separate covariates, which may have resulted in residual confounding. Future studies should include validated measures of sleep and chronotype to further clarify their roles in the relationship between daily behavioral structure and adolescent mental health. Fourth, the unexpected direction of the physical activity findings suggests that variable coding, activity context, and control of confounding require further refinement in future research. Future studies may adopt longitudinal designs in multischool and multiregion samples, incorporate accelerometry, screen logs, and sleep monitoring, and simultaneously include academic pressure, family support, and symptoms of depression and anxiety to more clearly characterize the relationship between daily behavioral structure and mental health in senior high school students.

## 5 Conclusion

Mental health among senior high school students was generally at a moderate level. Their daily behavioral profile was characterized by prolonged sedentary time, overall low physical activity, relatively regular main meals, and room for improvement in supplementary dietary habits. Mental health was positively associated with dietary habits and lifestyle and negatively associated with sedentary behavior, with sedentary behavior showing the most prominent association in both the correlation and regression analyses. Weekday lifestyle and regular meal habits retained stable positive associations in the multivariable models, and BMI was also associated with mental health. Although physical activity was significantly associated with mental health, the direction of this association should be interpreted cautiously in light of the measurement approach and study design. These findings suggest that mental health in senior high school is not only a psychological issue in itself, but is also closely related to the repeatedly accumulated structure of daily behaviors within the school context. For school health promotion, reducing prolonged uninterrupted sitting, optimizing weekday routines, maintaining regular main meals, and providing more targeted support in relation to students' physical status may better match the practical needs of senior high school students than a narrow focus on increasing exercise alone, and may also be more conducive to developing feasible mental health promotion strategies.

## Data Availability

The raw data supporting the conclusions of this article will be made available by the authors, without undue reservation.
